# Bowel Movement Frequency, Laxative Use, and Mortality From Coronary Heart Disease and Stroke Among Japanese Men and Women: The Japan Collaborative Cohort (JACC) Study

**DOI:** 10.2188/jea.JE20150123

**Published:** 2016-05-05

**Authors:** Yasuhiko Kubota, Hiroyasu Iso, Akiko Tamakoshi

**Affiliations:** 1 Public Health, Department of Social Medicine, Osaka University Graduate School of Medicine, Suita, Osaka, Japan; 1 大阪大学大学院医学系研究科社会医学公衆衛生学教室; 2 Department of Public Health, Hokkaido University Graduate School of Medicine, Sapporo, Japan; 2 北海道大学大学院医学系研究科社会医学講座公衆衛生学分野

**Keywords:** bowel movement frequency, laxatives, constipation, mortality, atherosclerosis, 排便回数, 下剤, 死亡リスク, 動脈硬化

## Abstract

**Background:**

The associations of bowel movement frequency and laxative use with cardiovascular disease (CVD) are unclear.

**Methods:**

A total of 72 014 subjects (29 668 men and 42 346 women) aged 40 to 79 years, without a history of CVD or cancer, completed a lifestyle questionnaire at baseline between 1988 and 1990 that included information on bowel movement frequency (daily, every 2–3 days, or once every 4 or more days) and laxative use (yes or no), and were followed-up until 2009.

**Results:**

During the subjects’ 1 165 569 person-years of follow-up, we documented 977 deaths from coronary heart disease (561 men and 416 women), 2024 from total stroke (1028 men and 996 women), 1127 from ischemic stroke (606 men and 521 women), and 828 from hemorrhagic stroke (388 men and 440 women). The prevalence of CVD risk factors, such as diabetes, stress, depression, and physical inactivity, was higher in laxative users and in those with a lower frequency of bowel movements. The multivariable HRs (95% confidence intervals [CIs]) of laxative users were as follows: 1.56 (95% CI, 1.21–2.03) for coronary heart disease and 1.37 (95% CI, 1.07–1.76) for ischemic stroke in men, and 1.27 (95% CI, 1.08–1.49) for total stroke, and 1.45 (95% CI, 1.17–1.79) for ischemic stroke in women. Similar results were observed even after the exclusion of deaths that occurred early in the follow-up period. A significant association between bowel movement frequency and mortality from CVD was not observed.

**Conclusions:**

Constipation could be a marker of exposure to CVD risk factors, and laxative use could be a risk factor for mortality from coronary heart disease and ischemic stroke.

## 要約

**背景：:**

排便回数・下剤使用と循環器疾患との関連は明らかになっていない。

**方法：:**

JACC studyで、循環器疾患およびがん既往のない40-79歳の男女72,014人（男性29,668人、女性42,346人）を1998-1990年から2009年まで追跡した。彼らはすべてベースライン時に排便回数（毎日、2-3日に1回、4日以上で１回）および下剤使用（はい、いいえ）に関する質問に回答している。

**結果：:**

1,165,596人年の追跡期間中、冠動脈疾患死亡997人（男性561人、女性416人）、全脳卒中死亡2,028人（男性1,028人、女性996人）、虚血性脳卒中1,127人（男性606人、女性521人）、および出血性脳卒中828人（男性388人、女性440人）であった。糖尿病、ストレス、うつ、および運動不足といった循環器疾患のリスクファクターの頻度は、下剤未使用者や排便回数の多い者に比べて、下剤使用者や排便回数の少ない者で低かった。下剤使用者の多変量調整ハザード（95%信頼区間）は、男性の冠動脈疾患死亡で1.56（1.21-2.03）、虚血性脳卒中で1.37（1.07–1.76）、女性の全脳卒中で1.27（1.08-1.49）、虚血性脳卒中で1.45（1.17-1.79）であった。早期死亡を除外した後も同様の結果が観察された。排便回数と循環器死亡との間には有意な関連は認めなかった。

**結語：:**

便秘は循環器疾患に危険因子曝露を示すマーカーである可能性がある。また、下剤使用は冠動脈疾患および虚血性脳卒中死亡の危険因子である可能性も示された。

## INTRODUCTION

There is growing awareness of a link between the gut and cardiovascular disease (CVD), particularly with respect to the progression of atherosclerosis,^1^^–^^3^ so cultivation of a more thorough understanding of this relationship is very important.

Constipation, one of the most common digestive disorders,^4^ is associated with lifestyle factors, such as stress and disturbed dietary habits. As such, constipation is expected to be closely linked to CVD. However, to the best of our knowledge, only one study has examined the association of constipation with CVD^5^; this study concluded that constipation, rather than being a risk factor for CVD itself, is a marker of exposure to CVD risk factors and increased CVD risk, specifically from atherosclerosis.^5^ This appears to be a reasonable conclusion, since many causes of constipation, such as aging and stress, are also risk factors for CVD. In addition to causing constipation, atherosclerosis could cause atherosclerotic diseases, such as coronary heart disease and stroke.^6^^–^^8^ However, this study included only women and used their perceived level of difficulty of having bowel movements, rather than bowel movement frequency, as an index of constipation. Therefore, this study’s findings require further confirmation among men and an investigation of other factors related to constipation.

In the present study, we investigated how a low frequency of bowel movements and the use of laxatives, which are characteristics often seen in constipated people, might influence mortality from coronary heart disease and stroke among Japanese men and women.

## METHODS

### Study population

The Japan Collaborative Cohort Study for the Evaluation of Cancer Risks (JACC study) was sponsored by the Ministry of Education, Sports, and Science and was initiated between 1988 and 1990 in 45 areas in Japan. A previous report described the details of this survey.^9^ Briefly, participants responded to self-administered questionnaires about their lifestyle and medical history regarding cancer and CVD. A total of 110 585 subjects (46 395 men and 64 190 women) aged 40 to 79 years old participated in JACC study. However, since the questionnaires in 12 of the 45 areas did not include information about the frequency of bowel movements and the use of laxatives, 23 524 subjects (10 431 men and 13 093 women) in these 12 areas were excluded from the present analysis. After this exclusion, 87 061 persons were eligible for participation in the present study. Of these, 76 174 participants (88%; 31 487 men and 44 687 women) completed questionnaires that included information on the frequency of bowel movements and the use of laxatives. In addition, 4160 subjects (1819 men and 2341 women) who had histories of cancer or CVD were further excluded because of the potential direct impact of these diseases on bowel movement and mortality. Ultimately, 72 014 subjects (29 668 men and 42 346 women) were included. The present study was approved by the ethics committees of the Osaka University Graduate School of Medicine and the Nagoya University School of Medicine.

### Main exposure: frequency of bowel movements and the use of laxatives

Participants were asked to provide information from the past year concerning the average frequency of their bowel movements and their laxative use (yes or no). Participants were grouped into the following three groups and then compared to examine the association of bowel movement frequency with CVD risks: those having a daily bowel movement (reference), those having a bowel movement every 2–3 days, and those having a bowel movement once every 4 or more days. Nonusers (reference) and users of laxatives were compared to examine the association of laxative use with CVD risk factors.

### Potential confounding factors

Potential confounding factors included age (years), sex (male or female), history of hypertension (yes or no), history of diabetes (yes or no), body mass index (sex-specific quintiles), alcohol intake (never, ex-drinker, or current drinker with an ethanol intake of 1–22, 23–45, 46–48, or ≥69 grams per day), smoking status (never, ex-smoker, or current smoker of 1–19 or ≥20 cigarettes per day), depressive symptoms (no symptoms, 1 symptom, or 2–4 symptoms),^10^ perceived mental stress (low, medium, or high), daily walking time (rarely, <30, 30–60, or >60 minutes per day), participation in sports (rarely, 1–2, 3–4, or ≥5 hours per week), dietary fiber intake (sex-specific quintiles), and menopausal status (pre- or postmenopausal). Participants were asked about average intake frequency of 40 food items without specifying portion size as follows: almost never, once or twice per month, once or twice per week, 3–4 times per week, and almost everyday.^11^ Intakes of foods and nutrients were calculated by the Japanese food composition table (4th edition), and standard portion sizes were derived from weighted dietary records.^11^ The value of dietary fiber intake obtained by enzymatic-gravimetric methods^12^ were derived from the food composition table.^11^ The intake of dietary fiber was adjusted for energy intake using the nutrient residual model.^13^ In addition, since geographical area has been reported to be associated with constipation and risk of cardiovascular disease,^14^^–^^16^ living in urban or rural areas was also included as a potential confounding factor. In this study, we defined cities in Japan (population ≥50 000) as urban areas and towns or villages (population <50 000) as rural areas.

### Mortality surveillance

Mortality surveillance was systematically performed by reviewing death certificates, which were sent to each public health center, and mortality data were centralized at the Ministry of Health and Welfare.^17^ The underlying causes of death were coded according to the International Classification of Diseases, 10th Revision (ICD-10). Deaths were confidently ascertained from death certificates at public health centers. The follow-up of subjects lasted until the end of 2009, or until death, whichever occurred first; exceptions were made for cases in which the follow-up was terminated in a study area, which occurred in four areas in 1999, four areas in 2003, and two areas in 2008. Follow-up endpoints included coronary heart disease, total stroke, ischemic stroke, and hemorrhagic stroke. Death from coronary heart disease was defined as ICD-10 codes I20 to I25, total stroke as I60–I69, ischemic stroke as I63 or I69.3, and hemorrhagic stroke as I60 to I62 or I69.0 to I69.2.

### Statistical analysis

We calculated sex-specific, age-adjusted prevalence and mean values of potential confounding factors and compared laxative use groups using χ^2^ tests or *t*-tests. For the three bowel movement frequency groups, we performed a test for trend using linear or logistic regression analysis. The person-years of follow-up from the baseline (1988 to 1990) to each endpoint (death, a move from the community, or the end of follow-up) were also calculated. Sex-specific hazard ratios (HRs) and their 95% confidence intervals (CIs) were computed for mortality outcomes after adjusting for age and other potential confounding factors using Cox proportional hazard models. The proportional hazard assumption in Cox regression was tested, and no violation was found. The SAS version 9.4 software (SAS Institute Inc., Cary, NC, USA) was used for statistical analyses. All statistical analyses were two-tailed, and *P* values <0.05 were considered significant.

## RESULTS

Table 1 lists the baseline characteristics according to bowel movement frequency and the use of laxatives. Compared to men with daily bowel movements or male nonusers of laxatives, those with less frequent bowel movements or those using laxatives tended to be older and leaner, drank less alcohol, and walked less, and were more likely to have a history of diabetes and high perceived mental stress, and have depressive symptoms. Women with less frequent bowel movements or those using laxatives were more likely to have a history of diabetes and high perceived mental stress, be current smokers, and live in urban areas; were less likely to take part in physical activities; and had lower intake of dietary fiber than those with daily bowel movement or female nonusers of laxatives. Both men and women using laxatives were more likely to have a history of hypertension than nonusers. In addition, men using laxatives were more likely to have frequent diarrhea than male nonusers of laxatives; on the other hand, female users of laxatives were less likely to have frequent diarrhea than female nonusers of laxatives.

**Table 1.** Age-adjusted baseline characteristics according to bowel movements frequency and use of laxatives

	Bowel movements frequency				Use of laxatives		
	
	Daily	Every 2–3 days	Every 4 or more days	*P* value	No	Yes	*P* value
Men							
Number at risk	26 346	3006	316	—	27 902	1766	—
Age, years	56.5	58.2	61.9	<0.001	56.3	63.3	<0.001
Body mass index, kg/m^2^	22.7	22.3	22.1	<0.001	22.7	22.4	<0.001
History of hypertension, %	20.1	17.2	16.9	<0.001	19.6	22.6	0.003
History of diabetes, %	6.1	9.3	10.7	<0.001	6.2	11.3	<0.001
Ethanol intake, g/day	34.2	30.3	29.7	<0.001	34.1	29.6	<0.001
Current smoker, %	54.1	55.6	63.8	0.002	54.4	52.8	0.195
High perceived mental stress, %	23.0	27.0	27.6	<0.001	22.9	32.5	<0.001
Two or more depressive symptoms, %	5.1	8.6	14.0	<0.001	5.3	9.4	<0.001
Walking ≥1 hour/day, %	50.1	43.1	34.6	<0.001	49.9	38.9	<0.001
Sports ≥5 hours/week, %	7.3	5.5	4.1	<0.001	7.1	6.8	0.656
Intake of dietary fiber^a^, g/d	12.5	12.5	12.3	0.335	12.5	12.7	0.195
Living in urban area, %	38.0	36.1	34.9	0.023	37.5	41.4	0.001
Bowel movement frequency every 4 or more days, %	—	—	—	—	6.2	80.5	<0.001
Use of laxatives, %	4.0	19.5	44.2	<0.001	—	—	—
Having frequent diarrhea, %	19.1	16.0	17.8	<0.001	18.7	21.4	0.008
Women							
Number at risk	28 738	11 747	1861		36 589	5757	
Age, years	57.4	56.2	56.4	<0.001	56.8	58.6	<0.001
Body mass index, kg/m^2^	23.1	22.6	22.5	<0.001	22.9	23.1	0.005
History of hypertension, %	22.5	20.3	18.2	<0.001	21.1	25.6	<0.001
History of diabetes, %	3.5	4.5	5.3	<0.001	3.6	6.0	<0.001
Ethanol intake, g/day	10.4	9.4	10.4	0.056	10.0	10.7	0.199
Current smoker, %	4.9	5.2	9.7	<0.001	4.5	9.1	<0.001
High perceived mental stress, %	19.5	21.9	28.0	<0.001	19.5	27.0	<0.001
Two or more depressive symptoms, %	7.1	9.3	13.8	<0.001	7.6	10.7	<0.001
Walking ≥1 hour/day, %	52.9	47.7	43.4	<0.001	52.1	44.4	<0.001
Sports ≥5 hours/week, %	4.8	3.8	2.8	<0.001	4.6	3.5	<0.001
Intake of dietary fiber^a^, g/d	12.4	12.0	11.7	<0.001	12.3	12.0	<0.001
Menopausal status, %	67.4	65.5	63.8	0.002	66.8	66.1	0.176
Living in urban area, %	39.5	40.6	41.0	0.026	38.7	47.5	<0.001
Bowel movement frequency every 4 or more days, %	—	—	—	—	2.6	15.6	<0.001
Use of laxatives, %	7.7	22.6	48.1	<0.001	—	—	—
Having frequent diarrhea, %	9.8	6.8	5.1	<0.001	9.0	6.7	<0.001

^a^Energy-adjusted value by nutrient residual model.

We documented 4604 deaths during the 1 165 569 person-years of follow-up for the 72 014 subjects (29 668 men and 42 346 women). Nine hundred and seventy-seven deaths resulted from coronary heart disease (561 men and 416 women), 2024 from total stroke (1028 men and 996 women), 1127 from ischemic stroke (606 men and 521 women), and 828 from hemorrhagic stroke (388 men and 440 women) (Table 2).

**Table 2.** Sex-specific, age-adjusted, and multivariable^a^ hazard ratios and 95% confidential intervals for cardiovascular mortality according to bowel movements frequency and use of laxatives

	Bowel movements frequency			Use of laxatives	
	
	Daily	Every 2–3 days	Every 4 or more days	No	Yes
Men					
Number at risk	26 346	3006	316	27 902	1766
Person-years	421 406	44 831	4092	447 244	23 085
Coronary heart disease, *n*	476	75	10	491	70
Age-adjusted HR (95% CI)	1.00	1.34 (1.05–1.71)	1.62 (0.87–3.04)	1.00	1.76 (1.36–2.27)
Multivariable HR (95% CI)	1.00	1.26 (0.99–1.62)	1.51 (0.80–2.83)	1.00	1.56 (1.21–2.03)
Total stroke, *n*	880	131	17	925	103
Age-adjusted HR (95% CI)	1.00	1.21 (1.01–1.46)	1.32 (0.82–2.13)	1.00	1.21 (0.98–1.48)
Multivariable HR (95% CI)	1.00	1.16 (0.96–1.40)	1.13 (0.70–1.83)	1.00	1.14 (0.92–1.41)
Ischemic stroke, *n*	510	83	13	530	76
Age-adjusted HR (95% CI)	1.00	1.28 (1.01–1.61)	1.60 (0.92–2.79)	1.00	1.41 (1.10–1.79)
Multivariable HR (95% CI)	1.00	1.23 (0.97–1.56)	1.40 (0.80–2.45)	1.00	1.37 (1.07–1.76)
Hemorrhagic stroke, *n*	341	45	2	367	21
Age-adjusted HR (95% CI)	1.00	1.14 (0.83–1.56)	0.46 (0.11–1.85)	1.00	0.73 (0.47–1.14)
Multivariable HR (95% CI)	1.00	1.10 (0.80–1.51)	0.39 (0.10–1.58)	1.00	0.68 (0.43–1.07)
Women					
Number at risk	28 738	11 747	1861	36 589	5757
Person-years	475 197	190 961	29 082	607 670	87 570
Coronary heart disease, *n*	288	106	22	334	82
Age-adjusted HR (95% CI)	1.00	1.04 (0.83–1.29)	1.40 (0.91–2.16)	1.00	1.42 (1.11–1.81)
Multivariable HR (95% CI)	1.00	0.98 (0.78–1.23)	1.25 (0.81–1.94)	1.00	1.28 (0.99–1.64)
Total stroke, *n*	702	248	46	805	191
Age-adjusted HR (95% CI)	1.00	0.99 (0.86–1.15)	1.19 (0.88–1.60)	1.00	1.37 (1.17–1.61)
Multivariable HR (95% CI)	1.00	0.98 (0.84–1.13)	1.13 (0.84–1.53)	1.00	1.27 (1.08–1.49)
Ischemic stroke, *n*	363	130	28	404	117
Age-adjusted HR (95% CI)	1.00	1.00 (0.82–1.22)	1.37 (0.93–2.01)	1.00	1.56 (1.27–1.92)
Multivariable HR (95% CI)	1.00	0.98 (0.80–1.20)	1.29 (0.87–1.90)	1.00	1.45 (1.17–1.79)
Hemorrhagic stroke, *n*	315	110	15	375	65
Age-adjusted HR (95% CI)	1.00	0.96 (0.78–1.20)	0.86 (0.51–1.45)	1.00	1.07 (0.82–1.40)
Multivariable HR (95% CI)	1.00	0.97 (0.78–1.20)	0.85 (0.50–1.43)	1.00	0.98 (0.75–1.28)

CI, confidential interval; HR, hazard ratio.

^a^Adjusted for age, history of hypertension, history of diabetes, body mass index, alcohol intake, smoking status, depressive symptoms, perceived mental stress, walking, sports, energy-adjusted dietary fiber intake, living in urban areas and menopausal status for women.

Age-adjusted and multivariable HRs were calculated for mortality from coronary heart disease, total stroke, and its subtypes, with respect to bowel movement frequency and the use of laxatives. Men having a bowel movement once every 2–3 days had a significantly higher risk of age-adjusted mortality from coronary heart disease, total stroke, and ischemic stroke compared to those with a daily bowel movement. However, these associations were no longer statistically significant after adjusting for potential confounding factors.

The use of laxatives was associated with higher risks of age-adjusted mortality from coronary heart disease and ischemic stroke in men and women, as well as from total stroke in women only. Further adjustment attenuated, but did not substantially change, these associations, except for the association of laxative use with mortality from coronary heart disease in women. For men, significant multivariable HRs were obtained for coronary heart disease (HR 1.56; 95% CI, 1.21–2.03) and ischemic stroke (HR 1.37; 95% CI, 1.07–1.76). For women, significant multivariable HRs were obtained for total stroke (HR 1.27; 95% CI, 1.08–1.49) and ischemic stroke (HR 1.45; 95% CI, 1.17–1.79).

To examine the possibility that a serious condition at baseline might have compelled people to use laxatives (reverse causation), we calculated the multivariable HRs for coronary heart disease, total stroke, and ischemic stroke after excluding deaths that occurred early in the study (Table 3). Similar results were observed even after the exclusion of deaths that occurred 1 to 7 years from the baseline.

**Table 3.** Multivariable^a^ hazard ratios and 95% confidential intervals for cardiovascular mortality according to use of laxatives after excluding deaths occurring within 1 to 7 years from baseline

	Men		Women	
	
Use of laxatives	No	Yes	No	Yes
Person-years	447 244	23 085	607 670	87 570
Coronary heart disease, *n*	491	70	334	82
Multivariable HR (95% CI)	1.00	1.56 (1.21–2.03)	1.00	1.28 (0.99–1.64)
Death within 1 year excluded, *n*	476	67	329	80
	1.00	1.58 (1.21–2.06)	1.00	1.28 (0.99–1.64)
Death within 2 years excluded, *n*	466	64	323	79
	1.00	1.56 (1.19–2.04)	1.00	1.30 (1.02–1.68)
Death within 3 years excluded, *n*	458	63	319	78
	1.00	1.58 (1.20–2.07)	1.00	1.31 (1.02–1.68)
Death within 4 years excluded, *n*	446	57	310	76
	1.00	1.48 (1.11–1.96)	1.00	1.33 (1.03–1.72)
Death within 5 years excluded, *n*	433	55	300	72
	1.00	1.48 (1.11–1.98)	1.00	1.32 (1.02–1.72)
Death within 6 years excluded, *n*	414	51	294	67
	1.00	1.48 (1.10–2.00)	1.00	1.27 (0.97–1.66)
Death within 7 years excluded, *n*	395	45	269	62
	1.00	1.43 (1.03–1.95)	1.00	1.31 (0.99–1.73)
Total stroke, *n*	925	103	805	191
Multivariable HR (95% CI)	1.00	1.14 (0.92–1.41)	1.00	1.27 (1.08–1.49)
Death within 1 year excluded, *n*	906	98	791	188
	1.00	1.12 (0.90–1.39)	1.00	1.28 (1.09–1.50)
Death within 2 years excluded, *n*	891	95	770	183
	1.00	1.12 (0.89–1.38)	1.00	1.29 (1.09–1.51)
Death within 3 years excluded, *n*	864	92	757	176
	1.00	1.11 (0.89–1.39)	1.00	1.27 (1.08–1.50)
Death within 4 years excluded, *n*	836	89	737	166
	1.00	1.12 (0.89–1.40)	1.00	1.24 (1.05–1.48)
Death within 5 years excluded, *n*	785	86	705	154
	1.00	1.20 (0.95–1.51)	1.00	1.23 (1.03–1.46)
Death within 6 years excluded, *n*	740	83	667	143
	1.00	1.26 (1.00–1.60)	1.00	1.22 (1.01–1.46)
Death within 7 years excluded, *n*	691	71	636	132
	1.00	1.21 (0.94–1.55)	1.00	1.20 (0.99–1.45)
Ischemic stroke, *n*	530	76	404	117
Multivariable HR (95% CI)	1.00	1.37 (1.07–1.76)	1.00	1.45 (1.17–1.79)
Death within 1 year excluded, *n*	524	74	401	116
	1.00	1.36 (1.06–174)	1.00	1.45 (1.18–1.79)
Death within 2 years excluded, *n*	519	73	398	115
	1.00	1.36 (1.06–1.76)	1.00	1.46 (1.18–1.80)
Death within 3 years excluded, *n*	506	71	395	111
	1.00	1.37 (1.06–1.77)	1.00	1.43 (1.15–1.77)
Death within 4 years excluded, *n*	495	69	389	107
	1.00	1.37 (1.05–1.77)	1.00	1.41 (1.13–1.75)
Death within 5 years excluded, *n*	470	66	381	101
	1.00	1.42 (1.09–1.86)	1.00	1.37 (1.10–1.72)
Death within 6 years excluded, *n*	446	66	362	96
	1.00	1.55 (1.19–2.03)	1.00	1.40 (1.11–1.75)
Death within 7 years excluded, *n*	423	58	351	89
	1.00	1.50 (1.13–1.99)	1.00	1.35 (1.07–1.71)

CI, confidential interval; HR, hazard ratio.

^a^Adjusted for age, history of hypertension, history of diabetes, body mass index, alcohol intake, smoking status, depressive symptoms, perceived mental stress, walking, sports, energy-adjusted dietary fiber intake, living in urban areas and menopausal status for women.

## DISCUSSION

We obtained two major findings in this prospective cohort study of Japanese men and women. First, those who had less frequent bowel movements or used laxatives had a higher prevalence of several previously identified risk factors for CVD, including diabetes, perceived mental stress,^18^ depression,^19^ and physical inactivity compared to those with more frequent bowel movements or nonusers of laxatives. Second, while bowel movement frequency was not associated with risk of mortality from CVD, laxative users had higher risks of mortality from coronary heart disease (men only), total stroke (women only), and ischemic stroke (men and women) compared to nonusers. To the best of our knowledge, this is the first study to investigate the association of bowel movement frequency or laxative use with mortality from CVD in an Asian population.

People with less frequent bowel movements or those using laxatives were more likely to be exposed to several risk factors for CVD. This result suggests that constipation could be a marker of exposure to CVD risk factors, which is compatible with the conclusions of a previous study.^5^

We found that the use of laxatives was associated with higher risk of mortality from coronary heart disease in men and from ischemic stroke in both men and women, even after adjusting for potential confounding factors. Several mechanisms for these associations could be considered. First, dehydration from the use of laxatives might have led to ischemic diseases because some kinds of laxatives soften defecation by preventing the gut from absorbing water. In fact, men using laxatives were more likely to have frequent diarrhea than male nonusers. On the other hand, women using laxatives were less likely to have frequent diarrhea than female nonusers, but this might be due to the possibility that their laxative-softened stools just did not look like diarrhea. Second, one kind of laxative has been reported to induce bacterial overgrowth and inflammation.^20^ The chronic use of this laxative might have destroyed gut flora, leading to chronic inflammation and finally the development of atherosclerosis. Third, serotonin might play a role in the associations observed in this study. Some laxatives increase the formation of serotonin,^21^ which causes vasoconstriction and increases smooth muscle cell aggregation.^22^^,^^23^ Therefore, laxatives might have increased the risks of mortality from ischemic diseases through the vasoconstrictive effect of serotonin. Fourth, the use of laxatives might be a marker of autonomic dysfunction and a predictor of cardiovascular disease from autonomic dysfunction. Autonomic dysfunction is associated with several disorders or diseases, including constipation,^24^ depression,^25^ hypertension,^26^^,^^27^ diabetes (diabetic neuropathy), and even CVD.^28^^–^^30^ In particular, several previous studies suggested that diabetic neuropathy could increase the risk of cardiovascular disease.^31^ Although autonomic nervous function was not measured in this study, we observed that people using laxatives were more likely to have a history of hypertension and diabetes and to have depressive symptoms at baseline, and these individuals had higher risk of mortality from CVD than nonusers. On the other hand, frequency of bowel movement was inversely associated with prevalence of history of hypertension in both men and women. Needing laxatives might reflect severe constipation and autonomic dysfunction more precisely than frequency of bowel movement, and use of laxatives may be a useful tool to predict future CVD events. Finally, since patients with a serious condition are generally more likely to use laxatives than healthier subjects, reverse causation is possible. However, even after the exclusion of deaths occurring within 7 years of the baseline, the association between the use of laxatives and mortality from ischemic diseases remained statistically significant. Therefore, the influence of reverse causation might be negligible.

The strengths of our study include its prospective design, the long follow-up duration, and the large number of participants. Nonetheless, some limitations need to be addressed. First, we had no detailed information on the frequency, amount, or the kind of laxatives used. Further research should use this information to confirm the cause of adverse effects associated with laxatives. Second, bowel movement frequency in this study was self-reported. Therefore, the possibility of misclassification of bowel movement frequency at baseline needs to be considered. Third, mortality data were used as endpoints, which may have led to misclassification of the diagnoses of cardiovascular diseases. However, previous studies have confirmed the validity of using death certificate diagnoses for these outcomes, given that the uses of computed tomography, magnetic resonance imaging, electrocardiography, and cardiac enzyme examination are widespread.^32^^,^^33^

In conclusion, constipation could be a marker of exposure to CVD risk factors among Japanese men and women, and the use of laxatives could be a risk factor for mortality from coronary heart disease and ischemic stroke. In clinical settings, constipation might be a useful tool for identifying patients at high risk for CVD, and the careful monitoring of laxative users might be necessary.

## ONLINE ONLY MATERIAL

Abstract in Japanese.
