# Supplementary material for: Bowel Movement Frequency, Laxative Use, and Mortality From Coronary Heart Disease and Stroke Among Japanese Men and Women: The Japan Collaborative Cohort (JACC) Study
Source: J Epidemiol. 2016 May 5;26(5):242–8. doi: 10.2188/jea.JE20150123 (PMC4848322; doi:10.2188/jea.JE20150123)
Supplement: Abstract in Japanese. [file je-26-242-s001.pdf]

日本人男女における排便回数・下剤使用と冠動脈疾患・脳卒中死亡との関連：JACC study

久保田　康彦[1]、磯　博康[1]、玉腰　暁子[2]、JACC Study Group
[1] 大阪大学大学院医学系研究科社会医学公衆衛生学教室
[2] 北海道大学大学院医学系研究科社会医学講座公衆衛生学分野

要約
背景：排便回数・下剤使用と循環器疾患との関連は明らかになっていない。
方法：JACC study で、循環器疾患およびがん既往のない 40-79 歳の男女 72,014 人（男性 29,668 人、女性 42,346 人）を 1998-1990 年から 2009 年まで追跡した。彼らはすべてベースライン時に排便回数（毎日、2-3 日に 1 回、4 日以上で 1 回）および下剤使用（はい、いいえ）に関する質問に回答している。
結果：1,165,596 人年の追跡期間中、冠動脈疾患死亡 997 人（男性 561 人、女性 416 人）、全脳卒中死亡 2,028 人（男性 1,028 人、女性 996 人）、虚血性脳卒中 1,127 人（男性 606 人、女性 521 人）、および出血性脳卒中 828 人（男性 388 人、女性 440 人）であった。糖尿病、ストレス、うつ、および運動不足といった循環器疾患のリスクファクターの頻度は、下剤未使用者や排便回数の多い者に比べて、下剤使用者や排便回数の少ない者で低かった。下剤使用者の多変量調整ハザード（95%信頼区間）は、男性の冠動脈疾患死亡で 1.56（1.21-2.03）、虚血性脳卒中で 1.37（1.07-1.76）、女性の全脳卒中で 1.27（1.08-1.49）、虚血性脳卒中で 1.45（1.17-1.79）であった。早期死亡を除外した後も同様の結果が観察された。排便回数と循環器死亡との間には有意な関連は認めなかった。
結語：便秘は循環器疾患に危険因子曝露を示すマーカーである可能性がある。また、下剤使用は冠動脈疾患および虚血性脳卒中死亡の危険因子である可能性も示された。

キーワード：排便回数、下剤、死亡リスク、動脈硬化
